# Alpha-Synuclein Proteins Promote Pro-Inflammatory Cascades in Microglia: Stronger Effects of the A53T Mutant

**DOI:** 10.1371/journal.pone.0162717

**Published:** 2016-09-13

**Authors:** Claire Hoenen, Audrey Gustin, Cindy Birck, Mélanie Kirchmeyer, Nicolas Beaume, Paul Felten, Luc Grandbarbe, Paul Heuschling, Tony Heurtaux

**Affiliations:** Life Sciences Research Unit, Laboratory of Neurobiology, University of Luxembourg, Faculty of Science, Technology and Communication, 7, avenue des Hauts Fourneaux, L-4362, Esch-sur-Alzette, Luxembourg; Universite Claude Bernard Lyon 1, FRANCE

## Abstract

Parkinson’s disease (PD) is histologically described by the deposition of α-synuclein, whose accumulation in Lewy bodies causes dopaminergic neuronal death. Although most of PD cases are sporadic, point mutations of the gene encoding the α-synuclein protein cause inherited forms of PD. There are currently six known point mutations that result in familial PD. Oxidative stress and neuroinflammation have also been described as early events associated with dopaminergic neuronal degeneration in PD. Though it is known that microglia are activated by wild-type α-synuclein, little is known about its mutated forms and the signaling cascades responsible for this microglial activation. The present study was designed to investigate consequences of wild-type and mutant α-synuclein (A53T, A30P and E46K) exposure on microglial reactivity. Interestingly, we described that α-synuclein-induced microglial reactivity appeared to be peptide-dependent. Indeed, the A53T protein activated more strongly microglia than the wild-type α-synuclein and other mutants. This A53T-induced microglial reactivity mechanism was found to depend on phosphorylation mechanisms mediated by MAPKs and on successive NFkB/AP-1/Nrf2 pathways activation. These results suggest that the microgliosis intensity during PD might depend on the type of α-synuclein protein implicated. Indeed, mutated forms are more potent microglial stimulators than wild-type α-synuclein. Based on these data, anti-inflammatory and antioxidant therapeutic strategies may be valid in order to reduce microgliosis but also to subsequently slow down PD progression, especially in familial cases.

## Introduction

Parkinson’s disease (PD) is one of the most common neurodegenerative diseases. This age-related disorder is clinically characterized by several motor symptoms such as bradykinesia, resting tremor and muscular rigidity, but also by non-motor symptoms as depression, gastrointestinal features and dementia [1,2]. PD is histologically described by the deposition of a physiological protein present in the brain, α-synuclein (α-syn). Its accumulation causes death of dopaminergic neurons resulting in the loss of the dopaminergic nigrostriatal pathway [3].

Αlpha-synuclein, a 140 amino-acid protein, accounts for about 1% of the total cytosolic proteins in brain. Its expression is highest in the dopaminergic neurons of the *substantia nigra pars compacta* (SNpc) and is intracellularly localized in presynaptic terminals. Therefore, α-syn might be involved in neurotransmitter release, synaptic vesicle trafficking, synaptic function and plasticity [4–7]. Alpha-synuclein proteins have the capacity to self-assemble, passing from unfolded monomers to oligomeric species, and then to heavy aggregates (called amyloid fibrils). The accumulation of these insoluble fibrils progressively promotes the formation of intracellular inclusions called Lewy bodies [4] within neurons and glial cells [8,9]. Recent studies suggest that α-synuclein oligomers are able to bind to lipids, disrupting cellular membrane integrity, and cause cell death both *in vitro* and *in vivo* [10–12]. Furthermore, it is known that aggregated α-synuclein present in Lewy bodies induces microglial activation leading to the death of dopaminergic neurons [13].

Although most of PD cases are sporadic, point mutations of *Snca*, the gene encoding the α-syn protein, cause inherited forms of PD [14]. In the late 20^th^ century, Polymeropoulos *et al*. described the first disease-causing mutation in different Mediterranean families. The substitution of alanine to threonine at position 53 of the α-syn protein (A53T) was identified as the cause of a severe autosomal dominant trait of Parkinsonism, characterized by an early onset with a short disease duration to death (less than 10 years). Afterwards, two other point mutations were identified, both responsible of dominant PD: A30P and E46K [15–17]. More recently, three additional familial mutations A53E, G51D and H50Q were identified [18–20].

Since the beginning of the century, some clues have demonstrated that α-syn can be secreted by cells and be present in biological fluids such as cerebrospinal fluid and blood plasma of both PD and healthy patients [21–23]. This extracellular α-syn may be responsible for the initiation and the maintenance of inflammatory events through the activation of microglia, the resident macrophages of the brain [24–26]. Activated microglia adopt morphological changes from a resting ramified shape to an amoeboid profile, accompanied by cell surface receptor impairments, production of reactive oxygen species (ROS) and release of cytokines [27–29]. Activated microglia phagocyte foreign antigens, proliferate and recruit additional microglial cells to mediate the inflammatory response. Although this phenomenon is essential to fight against infections or brain trauma, an over-activation can give rise to severe cellular damages and, in case of PD, could contribute to dopaminergic neuron depletion. The high levels of tumor necrosis factor alpha (TNFα) and interleukin-1beta (IL-1β) proteins assayed in the SNpc, striatum, cerebrospinal fluid and peripheral blood mononuclear cells from idiopathic patients reinforce this hypothesis [30]. The CXCL10 chemokine, also known as interferon gamma-induced protein 10 (IP-10), has also been found to be elevated in the CSF of brain patients and to be linked to neurodegeneration [31]. These three key inflammatory mediators (TNFα, IL-1β and CXCL10) have been described as markers associated with the microglial pro-inflammatory phenotype [32,33]. Given the importance of neuroinflammation in the development of PD, further studies are necessary to understand the precise role of α-synuclein in the microglial reactivity.

Therefore, we investigated whether different α-synuclein proteins were able to activate microglial cells and to promote subsequently a pro-inflammatory state. We provide a direct demonstration that the magnitude of the reactive microgliosis is higher after an A53T exposure compared to the wild-type protein or the other mutants. We also detail a large part of this A53T-induced microglial reactivity mechanism.

## Materials and Methods

### Ethics Statement

All animal experiments were carried out according to the 2010/63/EU European Union Directive and internal ethical committee regulations. The protocol was approved by the Animal Experimentation Ethics Committee (AEEC) of the University of Luxembourg. All efforts were made to minimize suffering. Newborn mice were decapitated.

### Cell cultures

Mixed glial cell cultures were prepared from newborn C57BL/6J mouse brains (Harlan, The Netherlands) as previously described [34]. Cells were plated and grown in Dulbecco’s Modified Eagle Medium (DMEM) supplemented with 10% fetal bovine serum (FBS), 100 U/ml penicillin and 100 μg/ml streptomycin at 37°C in a humidified atmosphere containing 5% CO_2_. The culture medium was changed after 3 days of culture. After 12-14 days, mixed glial cell cultures had reached confluence. Microglia present in the astrocyte monolayer were collected by a positive selection using a magnetic cell sorting (MACS) approach, as previously described [34]. Cells were selected using an anti-CD11b antibody following the manufacturer’s instructions (Miltenyi Biotec, The Netherlands). Finally, microglia were plated in a mix (1:1 v/v) of DMEM and mixed glial cell culture-conditioned medium at 37°C in a humidified atmosphere containing 5% CO_2_. Microglial cultures were treated 24 h later. Cell culture products were provided by Invitrogen (Belgium).

### Cell treatments

Purified recombinant human α-synucleins (wild-type and mutants proteins; AJ Roboscreen GmbH, Germany) were initially resuspended in sterile water at a concentration of 100 μM and stored at -20°C. Endotoxin contamination of the different α-synuclein aliquots was evaluated by the commercial PYROGENT™ Plus Gel Clot LAL Single Test Vials (Lonza, Belgium).

Microglial cultures were exposed to wild-type and mutant purified recombinant α-synucleins at a final concentration of 5 μM. Microglial cultures were also exposed to lipopolysaccharide (LPS 055:B5 from Escherichia coli, Sigma, Belgium) at 1 ng/ml or to menadione (10 μM, Sigma), a reactive oxygen generator. Cells were pre-treated for 1 h with 10 μM of MAPKs inhibitors (inhibitors of p38 (SB203580), ERK (PD98059) or JNK (SP600125)) before A53T treatment.

### Characterization of the different α-synuclein protein preparations

Wild-type and mutant α-synuclein proteins were characterized by gel electrophoresis. Sample buffer containing no SDS or β-mercaptoethanol was added to the different α-synuclein preparations. Alpha-synuclein proteins (0.25 μg) were resolved on a 12% acryl/bis-acrylamide gel in the absence of SDS (non-denaturating electrophoresis). All samples were electrophoresed using a Tris-Glycine-SDS buffer. Proteins were transferred to a 0.45 μm nitrocellulose membrane. The membrane was blocked in 3% (w/v) nonfat dry milk in phosphate-buffered saline (pH 7.4) containing 0.1% (v/v) Tween 20 for 1 h at room temperature. The blot was incubated overnight at 4°C with the 4D6 primary antibody (1:2000, Covance, USA), a mouse monoclonal antibody specific to α-synuclein proteins. For revelation by chemiluminescence, a peroxydase-conjugated anti-mouse antibody (Amersham Biosciences, UK) was applied at a 1:2000 dilution. Antibody complexes were detected by using SuperSignal West Femto Maximum Sensitivity Substrate (Pierce, Belgium) on a ChemiDoc™ XRS+ System (Bio-Rad, Belgium).

### Cellular viability assays

Mitochondrial function was assessed as an index of cellular viability using the MTT method. This assay measures the ability of mitochondrial dehydrogenases to convert 3-[4,5-dimethylthiazol-2-yl]-2,5-diphenyltetrazolium bromide (MTT) to colored insoluble formazan. Cells were seeded in 48-well plates at an initial density of 1 x 10^5^ cells/well. After treatment, cells were incubated for 3 h at 37°C with MTT (0.35 mg/ml) diluted in PBS. Then, the medium was removed, and cells were lysed by addition of DMSO. Absorbance was measured at 540 nm using a microplate reader (TECAN, Austria). Viability was estimated from the absorbance of treated versus untreated cells (100%).

Cell viability was also assessed with a cytotoxicity detection kit (Roche Applied Science, Germany) based on the measurement of LDH released from damaged cells. Cells were seeded on 48-well plates at an initial density of 1 x 10^5^ cells/well. According to the manufacturer’s instructions, culture supernatants were sampled after treatments and incubated for up to 30 min at room temperature with a freshly prepared reaction mixture (v/v). Absorbance was then determined at 490 nm using a microplate reader (TECAN). Viability was estimated from the absorbance of treated versus untreated cells (100%).

### Immunocytochemistry

Microglial cells were plated on poly-L-lysine coated coverslips. After treatments, cells were then fixed with paraformaldehyde (4% in PBS) for 20 min at room temperature, and permeabilized for 5 min in PBS containing 0.3% Triton X100. Following 3 washings in PBS, the blocking step was realized with 3% BSA in PBS at room temperature for 30 min. The cells were then incubated overnight at 4°C with a rabbit anti-Iba1 (1:300, Biocare Medical, USA). After washing steps with PBS, cells were incubated with Alexa488-conjugated anti-rabbit (1:1000, Life Technologies, Belgium) at room temperature for one hour. The cells were washed and mounted with Dapi-Fluoromount G (SouthernBiotech, USA). Cells were observed under a LSM 510 META inverted confocal microscope (Carl Zeiss Micro Imaging, Göttingen, Germany) at a 40-fold magnification.

### Real-Time reverse transcription-polymerase chain reaction (RT-PCR) analysis

Total RNA was isolated from microglia using the innuPREP RNA Kits (Westburg, The Netherlands) according to the manufacturer’s protocol. Complementary DNA (cDNA) was synthesized from RNA samples using the ImProm-II Reverse Transcription System (Promega, The Netherlands). PCR analyses were performed on a Bio-Rad iCycler (iQ5 Real-Time PCR Detection System, Bio-Rad) using iQ^TM^ SYBR Green Supermix (Promega). Primer sequences (Table 1) were designed using the Beacon Designer Software (Bio-Rad). Gene expression was analyzed using the comparative threshold cycle (C_t_) method. The target gene was normalized to the endogenous reference gene, *Rpl27* (a housekeeping gene coding for a ribosomal protein). The mRNA expression fold change was calculated using the expression 2^-ddCt^, where ddCt = (C_t, *target*_−C_t, *Rpl27*_)treated sample−(C_t, *target*_−C_t, *Rpl27*_)_control sample_.

**Table 1:** Sequences of the different real-time PCR primers.

Target gene	Accession number	Sequences
***Cxcl10***	NM_021274	for: TTCTGCCTCATCCTGCTG
		rev: AGACATCTCTGCTCATCATTC
***Nos2***	NM_010927	for: AGCCCTCACCTACTTCCTG
		rev: CAATCTCTGCCTATCCGTCTC
***Ptgs2***	NM_011198	for: GCCTGGTCTGATGATGTATGC
		rev: GAGTATGAGTCTGCTGGTTTGG
***Rpl27***	NM_011289	for: ACATTGACGATGGCACCTC
		rev: GCTTGGCGATCTTCTTCTTG
***Tnf***	NM_013693	for: GGTTCTGTCCCTTTCACTCAC
		rev: TGCCTCTTCTGCCAGTTCC

### PCR arrays analysis

Mouse Cytokines and Chemokines PCR arrays (Qiagen, The Netherlands) were performed following the manufacturer’s instructions. Firstly, reverse transcription was performed following the protocol detailed previously (*RT-PCR analysis* Section). Secondly, PCR components mix was prepared by mixing complementary DNA with RT^2^ SYBR Green Mastermix. PCR arrays analyses were performed on a Bio-Rad iCycler (iQ5 Real-Time PCR Detection System) using a 2-stage program provided by the manufacturer: 10 min at 95°C followed by 40 cycles, consisting of denaturation at 95°C for 15 sec and annealing/extension at 60°C for 60 sec. Analysis of gene expression was performed using the comparative threshold cycle (C_t_) method.

### Measurement of pro-inflammatory mediator releases

Cells were seeded on 12-well plates at an initial density of 7 x 10^5^ cells/well. The production of prostaglandins E_2_ (PGE_2_), TNFα and CXCL10 was measured in the culture medium after treatments. PGE_2_ and TNFα / CXCL10 release was respectively measured by a commercially available Enzyme ImmunoAssay kit (Assay Designs, USA) and a sandwich Enzyme Linked ImmunoSorbent Assay (R&D Systems, USA). Absorbance was then measured at 540 nm using a microplate reader (TECAN).

### Immunoblotting

Microglial cells were plated into 25 cm^2^ flasks. Total proteins were extracted with RIPA buffer (Pierce) and 1% protease / phosphatase inhibitor cocktail (Pierce). Nuclear extracts were prepared using a nuclear extract kit according to the manufacturer’s instructions (Active Motif, CA). Protein content was determined using the Bio-Rad Protein Assay Kit according to the manufacturer’s protocol.

Total proteins (20 μg) or nuclear proteins (10 μg) were separated through SDS-PAGE on a 12% gel and transferred to nitrocellulose membrane. Blots were incubated with a mouse anti-Stat1 (1:500, BD Biosciences, Belgium), rabbit anti-phospho-Stat1 (1:1000, Bioke, The Netherlands), rabbit anti-p38 (1:1000, Calbiochem, Belgium), rabbit anti-phospho-p38 (1:1000, Bioke), rabbit anti-ERK (1:20000, Sigma), rabbit anti-phospho-ERK (1:2000, Bioke), mouse anti-α-tubulin (1:5000, AbCam, UK), rabbit anti-p65 (1:200, Santa Cruz Biotechnologies, Germany), rabbit anti-c-Fos (1:1000, Bioke), rabbit anti-Nrf2 (1:1000, Bioke) and mouse anti-HDAC1 (1:2000, AbCam) antibody. After washing, membranes were incubated with an anti-rabbit or anti-mouse IgG-HRP antibody (1:2000, Amersham Biosciences, UK). Antibody complexes were detected by using SuperSignal West Femto Maximum Sensitivity Substrate (Pierce) on a ChemiDoc™ XRS+ System (Bio-Rad).

### Reactive Oxygen Species detection

Reactive Oxygen Species (ROS) were detected by the use of the ROS indicator, 6-carboxy-2',7'-dichlorodihydrofluorescein diacetate (carboxy-H2DCFDA, Life Technologies), which is a chemically reduced, acetylated form of fluorescein. By the activity of ROS within cells, this non-fluorescent probe was converted to a green-fluorescent molecule. The 2-methyl-1,4-naphthoquinone (menadione, 10 μM) was used as a positive control for ROS generation. This compound generates intracellular ROS (superoxide anions, hydrogen peroxide) at multiple cellular sites through redox cycling.

Microglial cells were plated on poly-L-lysine coated coverslips. As previously described (see Immunocytochemistry Section), cells were then fixed after treatments with paraformaldehyde (4%) and permeabilized. After a blocking step, cells were incubated with a rabbit anti-Iba1 (1:300). After washing steps, cells were incubated with Cy3-conjugated anti-rabbit (1:1000, Jackson Immuno-Research, UK). Cells were washed and then mounted with Dapi-Fluoromount G. The observation was performed under a LSM 510 META inverted confocal microscope (Carl Zeiss Micro Imaging, Göttingen, Germany) at a 40-fold magnification.

### GSH/GSSG ratio determination

Intracellular levels of reduced (GSH) and oxidized (GSSG) glutathione were determined after treatments in microglial cultures using the luminescence-based GSH/GSSG-Glo^TM^ assay (Promega). Cells were seeded on 12-well plates at an initial density of 7 x 10^5^ cells/well. According to the manufacturer’s protocol, intracellular glutathione levels were measured and quantified using a GSH standard curve. Luminescence was measured using a microplate reader (FLUOstar OPTIMA, BMG Labtech, Offenburg, Germany).

### Statistical analysis

Data are represented as mean ± standard error of the mean (SEM) from at least three independent experiments. As normality could not be assessed on such samples, multiple group comparisons were made using a nonparametric analysis of variance (Kruskal-Wallis test) followed by pairwise comparisons with Dunn’s correction. All statistical analyses were performed using GraphPad Prism software and differences with *p* values less than 0.05 were considered significant.

For PCR arrays, the HTqPCR package of bioconductor (R packages dedicated to biology) was used to complete the analysis. One of the HTqPCR data analysis allowed to tag C_t_ value as « OK », « undetermined » (C_t_ value above 35) or « unreliable » (C_t_ value outside the expected distribution, computed by HTqPCR) for each sample. Samples with less than 75% of value tagged as « OK » were removed from the dataset. Four normalization strategies (quantile, rank invariant, rank invariant scaled, and geometric mean) were applied to the data. Visual inspection of the normalization result through boxplot and comparison of two dispersion measures (coefficient of variation and standard deviation) lead to choose the quantile method to normalize data. The differentially expressed genes (DEG) list was generated using the Limma package. The DEG were selected when adjusted *p* value (Benjamini-Hochberg correction) < 0.05 and fold changes ≥ 2 criteria have been applied.

## Results

### Activation of microglial cells after α-synuclein proteins exposure

MTT and LDH assays were used to determine changes in viability following exposure of primary mouse microglia to α-synuclein preparations, LPS and other treatments. No loss of viability occurred (data not shown).

**Fig 1:**
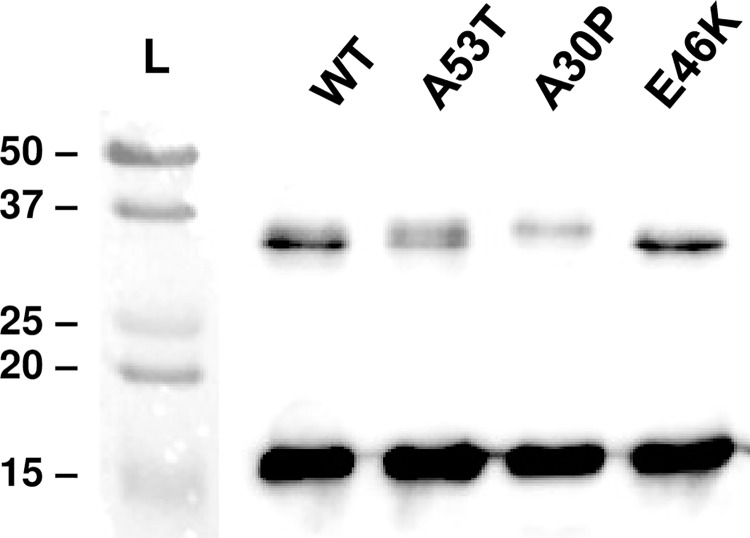
Analysis of α-synuclein preparations. Representative Western Blot of α-synuclein proteins (wild-type and mutants) separated by a 12% non-denaturing gel electrophoresis and probed with monoclonal antibody 4D6. Wild-type α-synuclein (WT), A53T, A30P and E46K mutant proteins were prepared at 2 μM. L = Ladder (kDa).

In this work, microglial cultures were exposed to the wild-type α-synuclein protein (WT, α-syn) as well as to three α-synuclein mutants (A53T, A30P and E46K). Based on LAL assays, no endotoxin contamination was detected in our different α-synuclein preparations. First of all, western-blot analysis (Fig 1) allowed us to visualize the size of these proteins. The different preparations contained exclusively monomer and dimer bands (respectively 15 and 30 kDa). The quantity of monomers of these four preparations was similar. Compared to WT and E46K preparations, which had equivalent amounts of dimers, the proportion of dimers was strongly lower in A30P (3-fold) and to a lesser extent in A53T preparation (2-fold).

**Fig 2:**
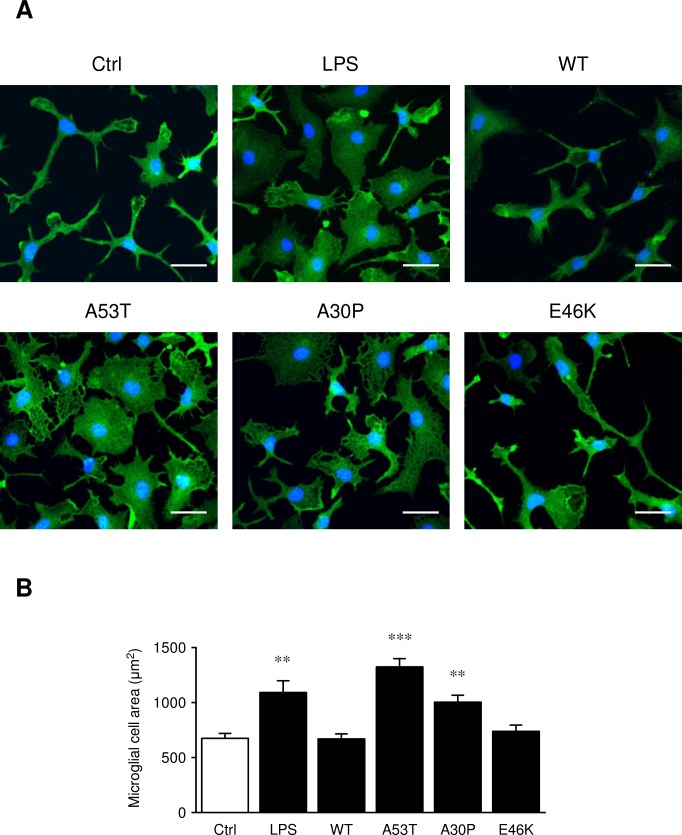
α-synuclein proteins alter differently the microglial cell morphology. Primary microglial cells were treated for 6 h with the pro-inflammatory compound LPS (positive control, 1 ng/ml), as well as with 5 μM of the wild-type α-synuclein (WT) and the 3 mutants (A53T, A30P, E46K) preparations (Fig 2A). Cells were labeled with the microglial marker Iba1 (green) and counterstained with nuclear stain DAPI (blue). Scale bar: 25 μm. After these treatments, microglial cell area (μm^2^) was measured (Fig 2B) with the open source image-processing package Fiji^®^ software. Data represent mean ± SEM of 20 measures per condition. ** *p* < 0.01, *** *p* < 0.001, significantly different from control condition.

To scale the α-synuclein protein effects, microglial cells were exposed to the pro-inflammatory compound (LPS, 1 ng/ml) for 6 h. The effects of the different proteins on microglia activation were first established by the changes of microglial morphology as observed by immunocytochemistry (Fig 2A). The microglial cell area (Fig 2B) was strongly increased after a LPS exposure (+ 62% compared to control condition) but also with the A53T peptide (+ 96%) and at a lower level with the A30P (+ 49%). Furthermore, no modification of morphology was observed with the wild-type α-syn protein (WT) and the E46K mutant. No significant change of cellular proliferation was observed in all these conditions (data not shown).

**Fig 3:**
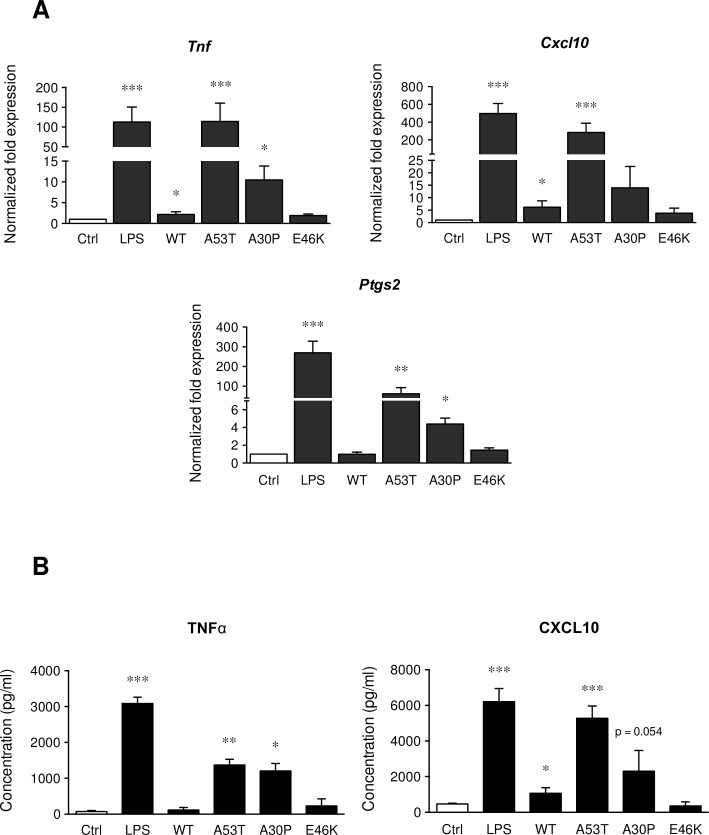
α-synuclein proteins modulate differently pro-inflammatory gene expressions as well as pro-inflammatory mediator releases. Following exposure of primary mouse microglial cells to LPS (1 ng/ml) and α-synuclein proteins (WT and mutants, 5 μM) for 6 h, pro-inflammatory gene expressions (*Tnf*, *Cxcl10* and *Ptgs2*) were analyzed by real-time PCR (Fig 3A). Control expression levels were fixed at 1.0. All gene expressions were normalized to *Rpl27* expression level. Results are given as mean ± SEM (n = 4 independent experiments). TNFα and CXCL10 protein releases (Fig 3B) were quantified by ELISA in supernatant of microglial cultures after 6 h of LPS (1 ng/ml) and α-synuclein proteins (5 μM) treatment. Results are given as mean ± SEM (n = 4 independent experiments). * *p* < 0.05, ** *p* < 0.01, *** *p* < 0.001, significantly different from control condition.

The effects of α-synuclein proteins exposure for 6 h were also assessed on pro-inflammatory gene expressions in microglial cells by real-time PCR (Fig 3A). Transcription levels of *Tnf*, *Cxcl10* and *Ptgs2* were strongly increased after a LPS exposure as well as after an A53T treatment. An A30P exposure was able to significantly increase *Tnf* and *Ptgs2* mRNA levels, but no significant modification was observed on the *Cxcl10* expression. The wild-type protein significantly increased *Tnf* and *Cxcl10* expression but at lower levels than after an A53T treatment. Moreover, an E46K exposure did not promote the up-regulation of these pro-inflammatory genes. As a result of *Tnf* and *Cxcl10* gene up-regulation, TNFα and CXCL10 releases increased significantly after 6 h of treatments (Fig 3B). A53T and A30P proteins similarly induced TNFα release whereas the CXCL10 release was higher after an A53T treatment than after an A30P exposure. The WT α-syn was not able to promote the release of TNFα but slightly increased the CXCL10 secretion. As observed at the mRNA expression levels, E46K was also not able to increase TNFα and CXCL10 levels in our conditions. Finally, the production of these pro-inflammatory proteins was lower after α-synuclein protein exposure than after a pro-inflammatory treatment (LPS).

Based on these results, the A53T protein appears to be a more potent activator compared to the other α-synuclein forms. This is why we decided to evaluate the A53T protein effects on additional pro-inflammatory cytokines / chemokines expression levels by PCR arrays. After an A53T exposure, 19 genes among the 84 assessed were noticed as significantly overexpressed (S1 Fig). *Tnf* and *Cxcl10* mRNA overexpression was confirmed, and the *Cxcl9* chemokine appeared to be the most up-regulated gene after an A53T exposure.

### A53T protein promotes ROS production in microglial cells

**Fig 4:**
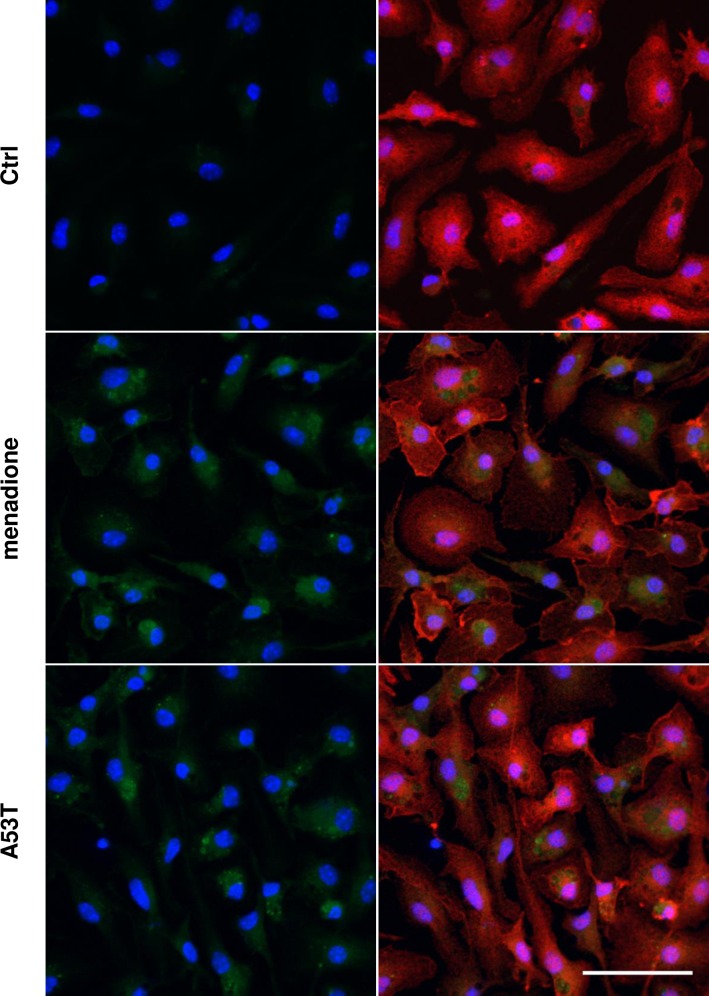
A53T protein promotes ROS production. Primary microglial cells were treated for 2 h with the A53T protein (5 μM) as well as with a reactive oxygen generator (menadione, 10 μM). After treatments, cells were incubated for 20 min with the 6-carboxy-2',7'-dichlorodihydrofluorescein diacetate (carboxy-H2DCFDA, 2 μM) probe. This nonfluorescent probe was converted to a green-fluorescent form by the activity of ROS within cells. Cells were also labeled with the microglial marker Iba1 (red) and counterstained with nuclear stain DAPI (blue). Scale bar: 50 μm.

The carboxy-H2DCFDA probe allowed visualization of intracellular reactive oxygen species (ROS) production (Fig 4). To evaluate the A53T protein-induced ROS production, microglial cells were exposed to menadione, a reactive oxygen generator. After 2 h of A53T exposure, microglial cultures were able to produce reactive oxygen species as shown by the increase of the intracellular green fluorescence. In these conditions, we also observed a significant decrease of the GSH/GSSG ratio at 24 h of treatment (Table 2) whereas no effect was observed at 6 h of treatment.

**Table 2:** An A53T exposure affects the GSH/GSSG balance. Primary microglial cells were treated for 6 h and 24 h with the A53T protein (5 μM) as well as with a reactive oxygen generator (menadione, 10 μM). After treatments, intracellular levels of reduced (GSH) and oxidized (GSSG) glutathione were measured. The ratio GSH/GSSG was established as a marker of oxidative stress. Results are given as mean ± SEM (n = 4 independent experiments).

	GSH/GSSG (6h)		GSH/GSSG (24h)	
	Mean (% of control)	SEM	Mean (% of control)	SEM
Control	100.0	10.6	100.1	6.4
Menadione	109.1	3.5	82.9 *	3.9
A53T	106.2	3.4	74.6 *	3.0

* *p* < 0.05, significantly different from control condition.

### Implication of JAK-STAT and MAPK signaling pathways in the A53T-induced microglial reactivity mechanism

**Fig 5:**
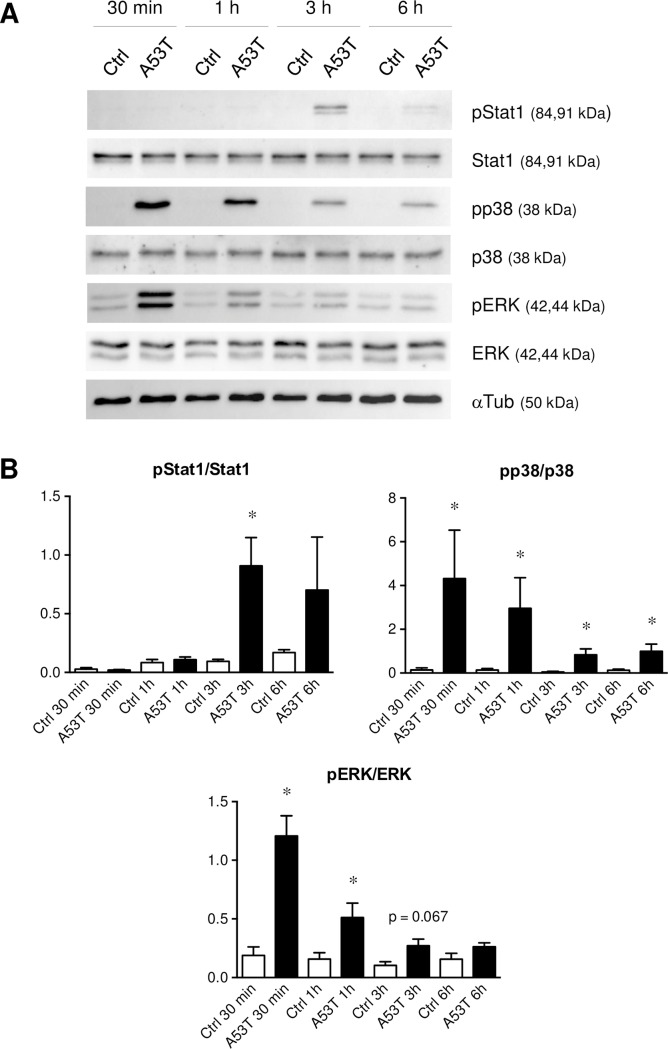
A53T protein induces STAT1 phosphorylation and MAPKs activation. After 30 min, 1 h, 3 h or 6 h of A53T protein exposure (5 μM), microglial cell lysates were subjected to western-blot analysis (Fig 5A) to determine the expression of phospho-Stat1 (pStat1), phospho-p38 (pp38) and phospho-ERK (pERK). α-Tubulin (αTub) was used as a loading control. These chemiluminescent detection assays were realized with 20 μg of total proteins. Ratios between phosphorylated versus non-phosphorylated proteins, called pStat1/Stat1, pp38/p38 and pERK/ERK, were described in Fig 5B. Results are given as mean ± SEM of at least three independent experiments. * *p* < 0.05, significantly different from control condition.

**Fig 6:**
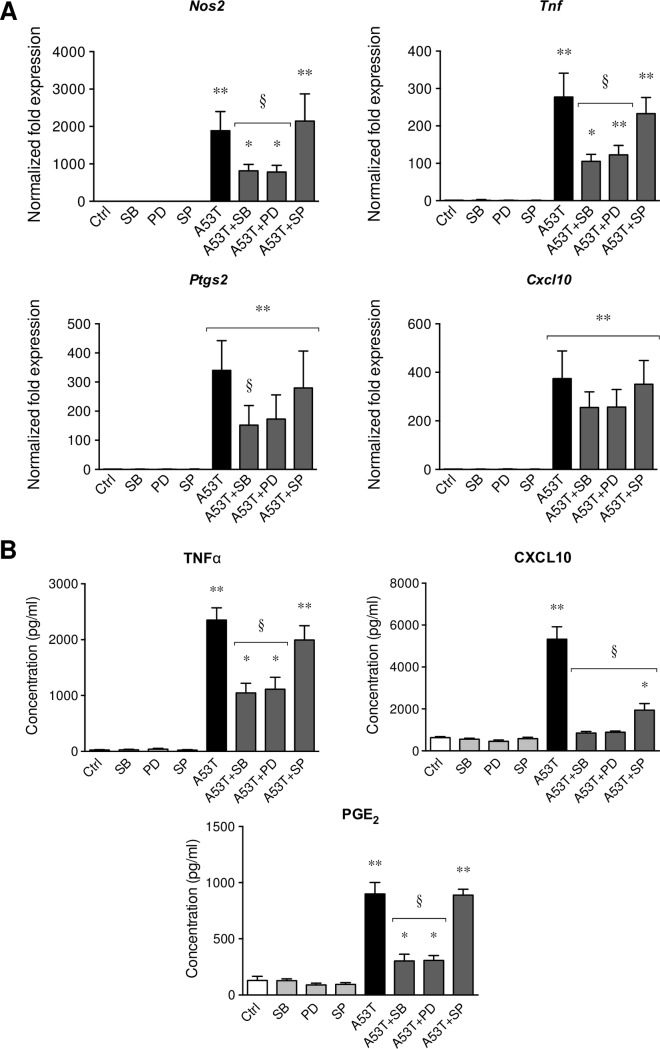
MAPKs are implicated in the A53T-induced microglial reactivity. Primary mouse microglial cell cultures were pre-treated for 1 h with 10 μM p38 inhibitor (SB203580, SB), ERK inhibitor (PD98059, PD) or JNK inhibitor (SP600125, SP) before the addition of A53T protein (5 μM) for 6 h. Pro-inflammatory gene expressions (*Nos2*, *Tnf*, *Ptgs2*, *Cxcl10*) were analyzed by real-time PCR (Fig 6A). Control expression levels were fixed at 1.0. All gene expressions were normalized to *Rpl27* expression level. Results are given as mean ± SEM (n = 5 independent experiments). Pro-inflammatory mediators released in the supernatant of microglial cultures (Fig 6B) were quantified by ELISA (TNFα, CXCL10) or EIA (PGE_2_). Results are given as mean ± SEM (n = 5 independent experiments). * *p* < 0.05, ** *p* < 0.01 significantly different from control condition; § *p* < 0.05 significantly different from A53T-treated cells.

In order to dissect the mechanisms underlying the A53T-induced microgliosis, total proteins were extracted after 30 min, 1 h, 3 h and 6 h of A53T exposure (Fig 5A and 5B). In these conditions, Stat1, p38 and ERK protein expressions were detectable and remained unchanged. Phospho-Stat1 (pStat1) expression was detected at 3 h of treatment and decreased at 6 h. As soon as 30 min of A53T treatment, phospho-p38 (pp38) and phospho-ERK (pERK) were overexpressed. These pp38 and pERK expressions decreased over time but appeared as still being up-regulated at respectively 6 h and 1 h. The expression of phospho-JNK (pJNK), another major actor of MAPK pathway, was not modulated in these conditions (data not shown). Based on these results, MAPK signaling appeared to be implicated in the inflammatory state induced by the A53T protein (Fig 5). We used subsequently different MAPK inhibitors (Fig 6) in order to confirm the implication of p38 (SB203580, SB), ERK (PD98059, PD) or JNK (SP600125, SP). The p38 and ERK inhibitors strongly decreased in the same manner the A53T-induced *Nos2* and *Tnf* overexpression by respectively 2.3-fold and 2.5-fold (Fig 6A). Contrary to the ERK inhibitor, the p38 inhibitor was able to significantly reduce the up-regulation of A53T-induced *Ptgs2* expression (2.1-fold). The JNK inhibitor had no effect on *Nos2*, *Tnf* and *Ptgs2* up-regulation. Moreover, none of these MAPK inhibitors were able to significantly modulate the up-regulation of *Cxcl10* mRNA expression. At the protein level (Fig 6B), p38 and ERK inhibitors strongly reduced TNFα, CXCL10 and PGE_2_ releases by respectively 2.2-fold, 6-fold and 2.9-fold. The JNK inhibitor had no effect on the A53T-induced TNFα and PGE_2_ releases but reduced by 2.7-fold the CXCL10 production.

### Involvement of the NFkB, AP-1 and Nrf2 pathways in the A53T-induced microglial reactivity mechanism

**Fig 7:**
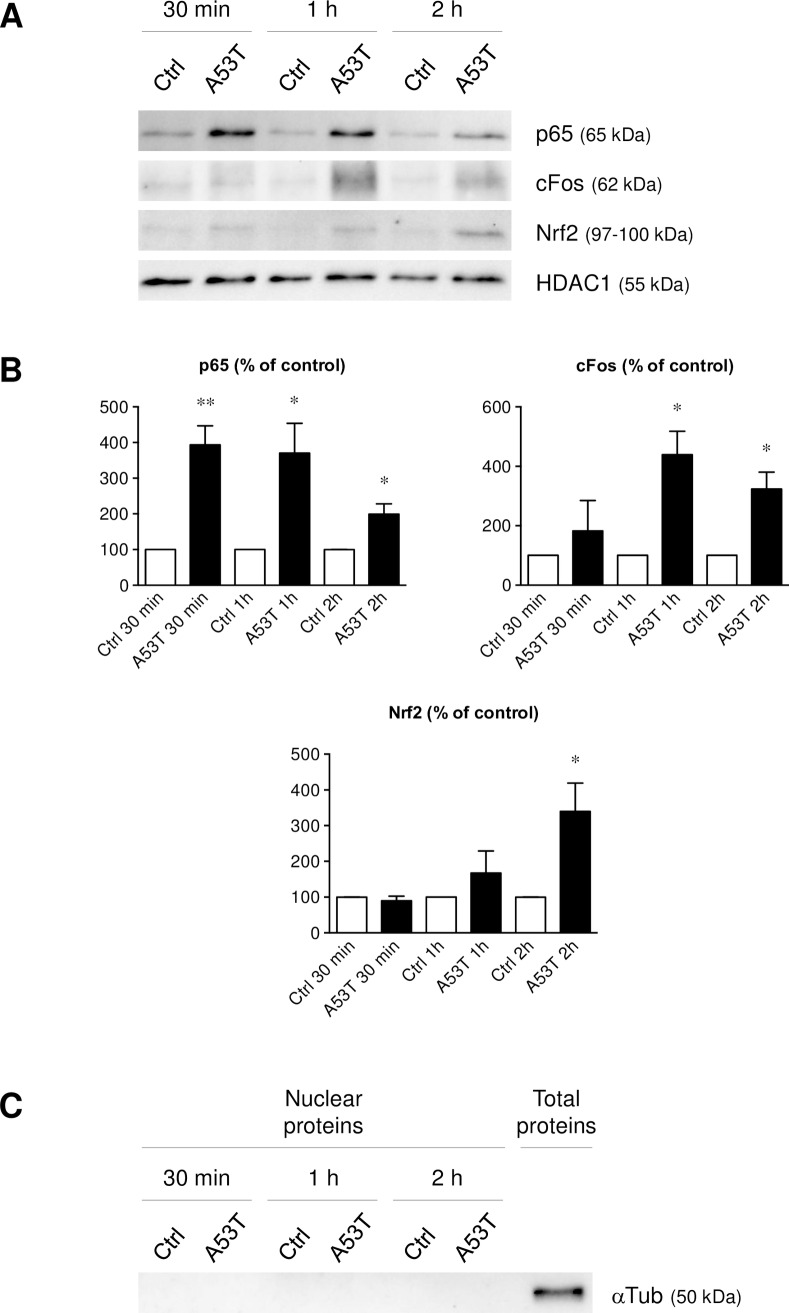
A53T protein induces NFkB, AP-1 and Nrf2 recruitment. Nuclear localization of the p65 subunit, c-Fos (subunit of AP-1) as well as Nrf2 was evaluated on microglial cells after a 5 μM A53T exposure for 30 min, 1 h or 2 h (Fig 7A). These chemiluminescent detection assays were realized by western-blot with 10 μg of nuclear proteins. The histone deacetylase 1 (HDAC1) was used as a loading control. Furthermore, p65, c-Fos and Nrf2 detected proteins were normalized and quantified (Fig 7B). Results are given as mean ± SEM of at least three independent experiments. * *p* < 0.05, ** *p* < 0.01, significantly different from control condition. A cytoplasmic protein negative control western-blot (αTub) was also realized (Fig 7C) to demonstrate the effectiveness of the nuclear isolation.

The two transcription factors, NFkB and AP-1, are known to be implicated in numerous inflammatory states. The nuclear factor (erythroid-derived 2)-like 2 (Nrf2) regulates cellular responses against oxidative and inflammatory stress. Therefore, NFkB, AP-1 and Nrf2 were investigated and quantified (Fig 7A and 7B). The effectiveness of the nuclear isolation was also verified (Fig 7C). Nuclear proteins were extracted after 30 min, 1 h and 2 h of A53T exposure (Fig 7A and 7B). After 30 min, NFkB activation was detected in microglial cells, evidenced by the nuclear localization of p65. This NFkB activation decreased over time but was still observed at 1 h and 2 h of treatment. Likewise, AP-1 activation was observed in our conditions by an increase of c-Fos subunit in the nucleus. Compared to NFkB activation, the AP-1 recruitment took place later, at 1 h and decreased at 2 h of A53T exposure. Furthermore, no detectable Nrf2 nuclear translocation could be observed in microglial cultures after 30 min and 1 h of A53T treatment, but only at 2 h of treatment. The decrease of the transcription factors recruitment coincided with a time-dependent decrease of the pro-inflammatory gene overexpression (S2 Fig).

## Discussion

Evidences of an ongoing inflammatory state in Parkinson’s disease come from studies showing increased pro-inflammatory molecules levels in the blood and cerebrospinal fluid (CSF) in patients [35,36]. Indeed, it is now well documented that pro-inflammatory molecules (cytokines, chemokines, prostaglandins) and reactive oxygen species are associated with neurodegeneration and particularly with Parkinson’s disease pathogenesis progression [37,38]. PD is histologically described by the deposition of α-synuclein and as a result by the accumulation of protein aggregates called Lewy bodies. Although most PD cases are sporadic, point mutations of the gene encoding the α-synuclein protein, cause inherited forms of PD.

Microglia are known to react rapidly and strongly to brain insults. These cells are clearly recognized as a principal player for neuroinflammation in the central nervous system [39,40]. In this work, based on *in vitro* experiments on primary murine microglia, we have established that microglia activation intensity appears to be α-synuclein protein-dependent. Indeed, the wild-type α-synuclein (WT α-syn) and the 3 corresponding mutants (A53T, A30P and E46K) differentially activate microglial cultures. Recent works have been published on the degree of α-synuclein-mediated toxicity, with regard to the conformation of the protein [4]. Here, we focussed our attention on monomers and dimers of α-synuclein that might correspond to an early stage of PD pathology.

This study is based on recombinant α-synuclein proteins. It is generally assumed that endogenous α-synuclein involved in Parkinson’s disease may be subject to post-translational modifications (phosphorylation, oxidation, nitrosylation, glycation or glycosylation) [41,42]. These modifications could strongly modify the pathogenic properties of the disease-associated proteins. This is not the case for recombinant proteins. Thus, a disparity of microglial activation could appear when comparing endogenous and recombinant α-synuclein exposure. A special care should be taken before issuing conclusions.

In the presence of recombinant α-synuclein proteins, our data showed an increase of the pro-inflammatory state. Indeed, after only 6 h of treatment, A53T and A30P mutants were able to modify microglia morphology, unlike WT α-synuclein and E46K exposures. Furthermore, the A53T mutant was also able to strongly overexpress *Tnf*, *Cxcl10* and *Ptgs2* mRNA levels, whereas WT α-syn or A30P only show a weak up-regulation of these pro-inflammatory genes. These mRNA expression level modulations confirm our data on cell morphology. TNFα and CXCL10 releases (Fig 3B) corroborate our pro-inflammatory gene expression analyses (Fig 3A). Furthermore, compared to a pro-inflammatory positive control (LPS treatment), an A53T exposure increases more weakly mRNA and protein expression levels. Finally, the A53T protein promotes a more pronounced pro-inflammatory phenotype on primary microglia and appears to be a more potent activator compared to the A30P mutant and the wild-type α-synuclein. The E46K protein has no effect on microglial activation (A53T > A30P > WT > E46K). These results are in accordance with other studies that have described a higher pro-inflammatory potential of the α-synuclein mutant proteins compared to the wild-type protein [43–45]. Data from the literature also describe that microglial cell lines often respond differently to pathological stimuli (e.g. synuclein or beta-amyloid protein) [46,47] and confirm the importance of working on primary cultures.

The conformation of the α-synuclein protein (monomers / oligomers / fibrils), responsible for toxicity in PD, is still not clear. Several works have described higher cytotoxicity of the soluble oligomers compared to fibrils [12,48,49]. However, many recent reports have described that α-syn fibrils could be toxic by inducing apoptotic cell death [50,51]. In our conditions, α-synuclein proteins are composed of monomers and dimers. Interestingly, WT and E46K proteins, the two preparations with more dimeric fragments (30 kDa), exhibit a less strong microglial reactivity compared to A53T and A30P. Moreover, the fact that these cells react more strongly to the presence of mutated α-synuclein, could lead to an earlier onset and faster progression of the pathology, as observed during familial PD forms [52,53]. Due to the composition of our α-synuclein preparations, it is important to note that the specific involvement of monomers, or dimers, on microglial reactivity could not be clearly determined. In additional experiments, we have also established that mutated proteins could have a higher ability to aggregate compared to the wild-type α-synuclein protein (data not shown). Using these recombinant proteins, it would be important to perform further experiments in order to verify the direct impact of monomers, dimers, as well as larger α-synuclein samples (oligomers, proto-fibrils, fibrils) on microglial reactivity.

A53T, A30P and E46K mutants, as well as wild-type α-synuclein, are natively unfolded under physiological conditions [17,54]. Sahay and collaborators described that the overall structure of the α-syn protein is not affected by these mutations [55]. However, A53T, A30P and E46K proteins present mutations in the N-terminal part of the α-synuclein protein. This N-terminal amphipathic region is important for α-synuclein functions and membrane interactions [56,57] as well as for macrophage activation [58]. Microglial cells are known to react to the presence of foreign substances by the expression of receptors called pattern recognition receptors (PRRs) on the cell surface. These PRRs include Toll-like receptors (TLRs), receptor for advanced glycation end products (RAGE) and scavenger receptors [59–61]. Ligation to PRRs triggers a succession of cellular signaling events, including the activation of signal transduction pathways [62–66]. Thus, a modification of the N-terminal region of the α-synuclein proteins may disrupt or facilitate the recognition and the binding of the protein on the microglial cell surface and consequently may modulate the activation of signaling pathways.

The signal transduction of the A53T protein-induced microglial reactivity was subsequently evaluated. First of all, the A53T protein was found to activate microglia in a time-dependent (S2 Fig) but also in a dose-dependent manner (S3 Fig). Then, by western-blot analyses, we have highlighted the activation of the Stat1 protein by the presence of phosphorylated Stat1 (pStat1) after an A53T exposure. The overexpression of pStat1, a marker expressed by reactive microglia, confirms the establishment of a pro-inflammatory state in these experimental conditions. We have also demonstrated the recruitment of activated p38 and ERK MAPKs, but no modulation of the JNK pathway has been revealed. Indeed, the use of MAPK inhibitors strongly decreases the A53T-induced microglial reactivity by reducing pro-inflammatory gene overexpression but also pro-inflammatory molecule releases. These results confirm that p38 and ERK MAPKs are implicated in the A53T-induced microglial activation. In the literature, *in vitro* and *in vivo* experiments confirm the implication of protein kinases in glial cells in neurodegenerative diseases [47,67]. ERK activation has also been described to act downstream of Stat signaling during microglial activation [68,69] and therefore confirms our results.

In this work, the A53T-induced microglial reactivity is associated with the recruitment of the NFkB, AP-1 and Nrf2 transcription factors. It is well established that many pro-inflammatory genes contain functional NFkB and/or AP-1 response elements. Our data on nuclear protein extracts reveal the recruitment of p65 (a NFkB subunit) in a time-dependent manner with a maximum reached after 30 min. The A53T protein also increases c-Fos (an AP-1 subunit) protein levels with a peak expression after 1 h of treatment. Several studies confirm our findings [70–72]. In many cell types, NFkB and AP-1 activities are known to be MAPK-dependent [47,73–75]. On the one hand, the NFkB activation has been shown to be MAPK-dependent by the phosphorylation of the NFkB inhibitory protein IkB [76,77]. On the other hand, AP-1 subunits have also been described to be phosphorylated by MAPKs on specific sites that strengthen their transcriptional activities [78].

A battery of genes encoding detoxification enzymes also contains antioxidant response element (ARE) sequences. This ARE is activated through the binding of its transcription factor, Nrf2. The Nrf2-ARE pathway is known to regulate the cell response against oxidative and inflammatory stress [79]. Lastres-Becker and collaborators have demonstrated that Nrf2 was able to modulate microglial reactivity [80]. The Nrf2 pathway is the major regulator of cytoprotective responses to endogenous and exogenous stresses caused by reactive oxygen species (ROS). In our conditions, an A53T exposure is able to increase the production of ROS after 2 h of treatment. The Nrf2 recruitment, after 2 h of A53T exposure, coincides with this increase of ROS production by microglia. Likewise, we have determined that an A53T exposure for 24 h was able to decrease the microglial GSH/GSSG ratio due to an increase of the GSSG level consequently to a ROS-induced GSH oxidation.

In summary, this work provides a good description of how the presence of A53T mutant protein can rapidly and strongly activates microglial cells by a mechanism in which MAPKs, NFkB, AP-1 and Nrf2 transcription factors are engaged. Our data suggest that NFkB and AP-1 activation are early and direct events of A53T signaling. The Nrf2 activation may be a secondary event that participates in both reducing ROS production but also providing a negative feedback control of inflammation [80–82]. The pro-inflammatory profile acquired by microglial cells, highlighted by our data, confirm the existence of a vicious circle that happens during PD progression [83]. These results are in accordance with the hypothesis that in early stages of PD, small diffusible α-synuclein proteins activate microglia leading to an inflammatory state [25]. Inflammatory stimuli, together with persistent microglial activation, perpetuate an activated state that potentiates neuronal death. More precisely, activation of microglia and release of numerous pro-inflammatory compounds play important roles in dopaminergic neuronal death in the *substantia nigra*, leading notably to the release of ATP but also of α-synuclein proteins, which again can both stimulate microglia [39,71,84].

The role of microglial cells in Parkinson’s disease remains still complex and unclear. However, our results reinforce the hypothesis that controlling neuroinflammatory mechanisms appear to be a useful strategy to contain PD progression. Therefore, these findings may be relevant to neurodegenerative diseases involving inflammation, but also to aging where brain metabolism can be deeply altered. Like in other neurodegenerative diseases, aging is one of the most significant risk factor for PD. Recently, Bliederhaeuser and collaborators have highlighted that age-related microglial alterations (increase of the inflammatory status, decrease of the phagocytic activity and of the antioxidant defenses) may contribute to an increased susceptibility to pathogens or abnormally folded proteins in neurodegenerative diseases [85]. Further studies are needed to fully understand the underlying mechanisms of dysregulated microglia with aging and their contribution to the progression of PD.

## Supporting Information

S1 Fig A53T protein up-regulates many pro-inflammatory gene expressions. Pro-inflammatory gene expression levels were analyzed by PCR arrays after 6 h of A53T exposure (5 μM). PCR arrays (RT^2^ Profiler™ PCR Array Mouse Cytokines & Chemokines, Qiagen, The Netherlands) were performed on 1 μg of total RNA per array plate and allowed to assess 84 different genes simultaneously. The differentially expressed genes after the A53T stimulation were listed when the criteria fold changes > 2 and *p* value < 0.05 have been applied. Control expression levels were fixed at 1.0. All gene expressions were normalized to housekeeping genes expression level. Results are given as mean ± SEM (n = 3 independent experiments).
(TIF)

S2 Fig The A53T exposure-induced microgliosis decreases over time. Pro-inflammatory gene expressions (*Nos2*, *Tnf* and *Ptgs2*) were analyzed by real-time PCR following exposure of primary mouse microglial cells to A53T protein (5 μM) for 6, 24 and 48 h. Control expression levels were fixed at 1.0. All gene expressions were normalized to *Rpl27* expression level. Results are given as mean ± SEM (n = 3 independent experiments).
(TIF)

S3 Fig A53T protein modulates pro-inflammatory gene expressions as well as pro-inflammatory mediators in a concentration-dependent manner. Following exposure of primary mouse microglial cells to different concentrations of A53T protein for 6 h, pro-inflammatory gene expressions (*Nos2*, *Tnf*, *Ptgs2* and *Cxcl10*) were analyzed by real-time PCR (S3A Fig). Control expression levels were fixed at 1.0. All gene expressions were normalized to *Rpl27* expression level. Results are given as mean ± SEM (n = 3 independent experiments). In these conditions, pro-inflammatory mediators released in the supernatant of microglial cultures were quantified by ELISA (S3B Fig). Results are expressed as fold increase compared to control and are given as mean ± SEM (n = 3 independent experiments). * *p* < 0.05, ** *p* < 0.01, significantly different from control condition.
(TIF)

## Abbreviations

α-syn: alpha-synuclein
AP-1: activator protein 1
ARE: antioxidant response element
cDNA: complementary DNA
C_t_: threshold cycle
CXCL10: C-X-C motif chemokine 10
Dapi: 4',6'-diamidino-2-phenylindole
DMEM: Dulbecco’s Modified Eagle’s Medium
ERK: extracellular signal-regulated kinase
FBS: fetal bovine serum
GSH: reduced glutathione
GSSG: oxidized glutathione
HDAC1: histone deacetylase 1
HRP: horseradish peroxidase
Iba-1: ionized calcium-binding adapter molecule 1
IkB: inhibitor of kappa B
IL-1β: interleukin-1β
JNK: c-Jun N-terminal kinase
LDH: lactate deshydrogenase
LPS: lipopolysaccharide
MACS: magnetic cell sorting
MAPK: mitogen-activated protein kinase
NFkB: nuclear factor-kappa B
Nos2: Nitric oxide synthase 2 (inducible)
PBS: phosphate buffered saline
PD: Parkinson’s disease
PGE_2_: prostaglandin E2
PRR: pattern recognition receptor
pStat1: phosphorylated Stat1
Ptgs2: prostaglandin-endoperoxide synthase 2
RAGE: receptor for advanced glycation end product
ROS: reactive oxygen species
RT-PCR: Real-Time polymerase chain reaction
SDS: sodium dodecyl sulfate
SNpc: Substantia Nigra pars compacta
Stat1: signal transducers and activators of transcription
TLR: Toll-like receptor
TNFα: Tumor Necrosis Factor α
WT: wild-type
