# Supplementary figures and images for: Alpha-Synuclein Proteins Promote Pro-Inflammatory Cascades in Microglia: Stronger Effects of the A53T Mutant
Source: PLoS One. 2016 Sep 13;11(9):e0162717. doi: 10.1371/journal.pone.0162717 (PMC5021287; doi:10.1371/journal.pone.0162717)

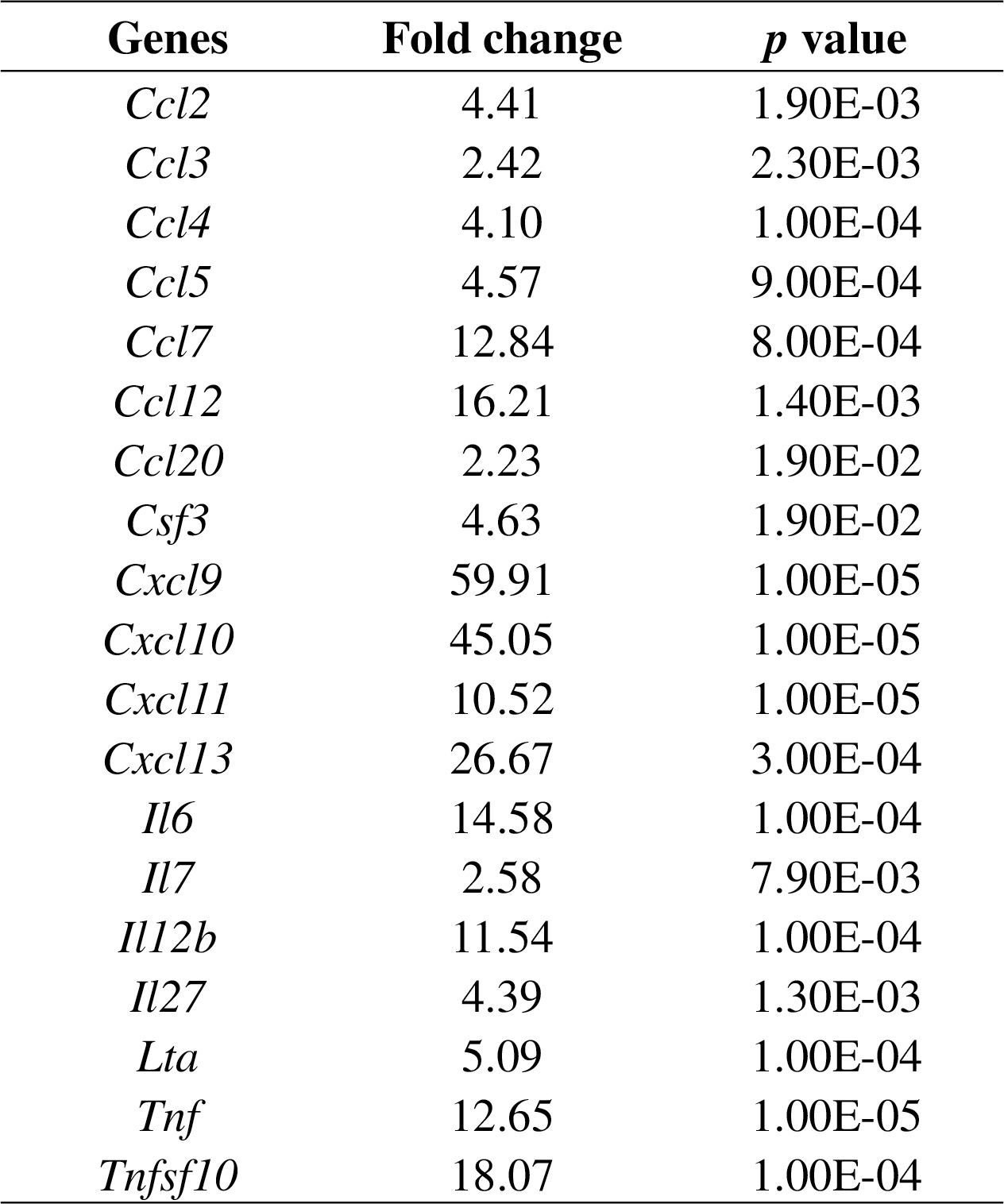

Supplement: S1 Fig — Pro-inflammatory gene expression levels were analyzed by PCR arrays after 6 h of A53T exposure (5 μM). PCR arrays (RT2 Profiler™ PCR Array Mouse Cytokines & Chemokines, Qiagen, The Netherlands) were performed on 1 μg of total RNA per array plate and allowed to assess 84 different genes simultaneously. The differentially expressed genes after the A53T stimulation were listed when the criteria fold changes > 2 and p value < 0.05 have been applied. Control expression levels were fixed at 1.0. All gene expressions were normalized to housekeeping genes expression level. Results are given as mean ± SEM (n = 3 independent experiments). (TIF) [file pone.0162717.s001.tif]

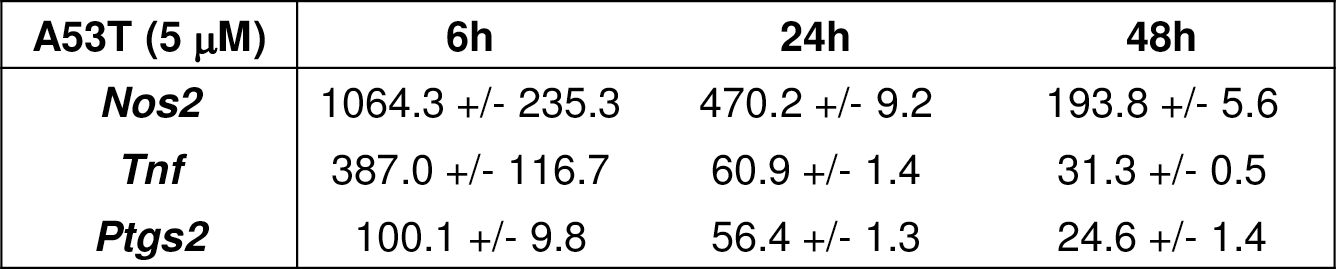

Supplement: S2 Fig — Pro-inflammatory gene expressions (Nos2, Tnf and Ptgs2) were analyzed by real-time PCR following exposure of primary mouse microglial cells to A53T protein (5 μM) for 6, 24 and 48 h. Control expression levels were fixed at 1.0. All gene expressions were normalized to Rpl27 expression level. Results are given as mean ± SEM (n = 3 independent experiments). (TIF) [file pone.0162717.s002.tif]

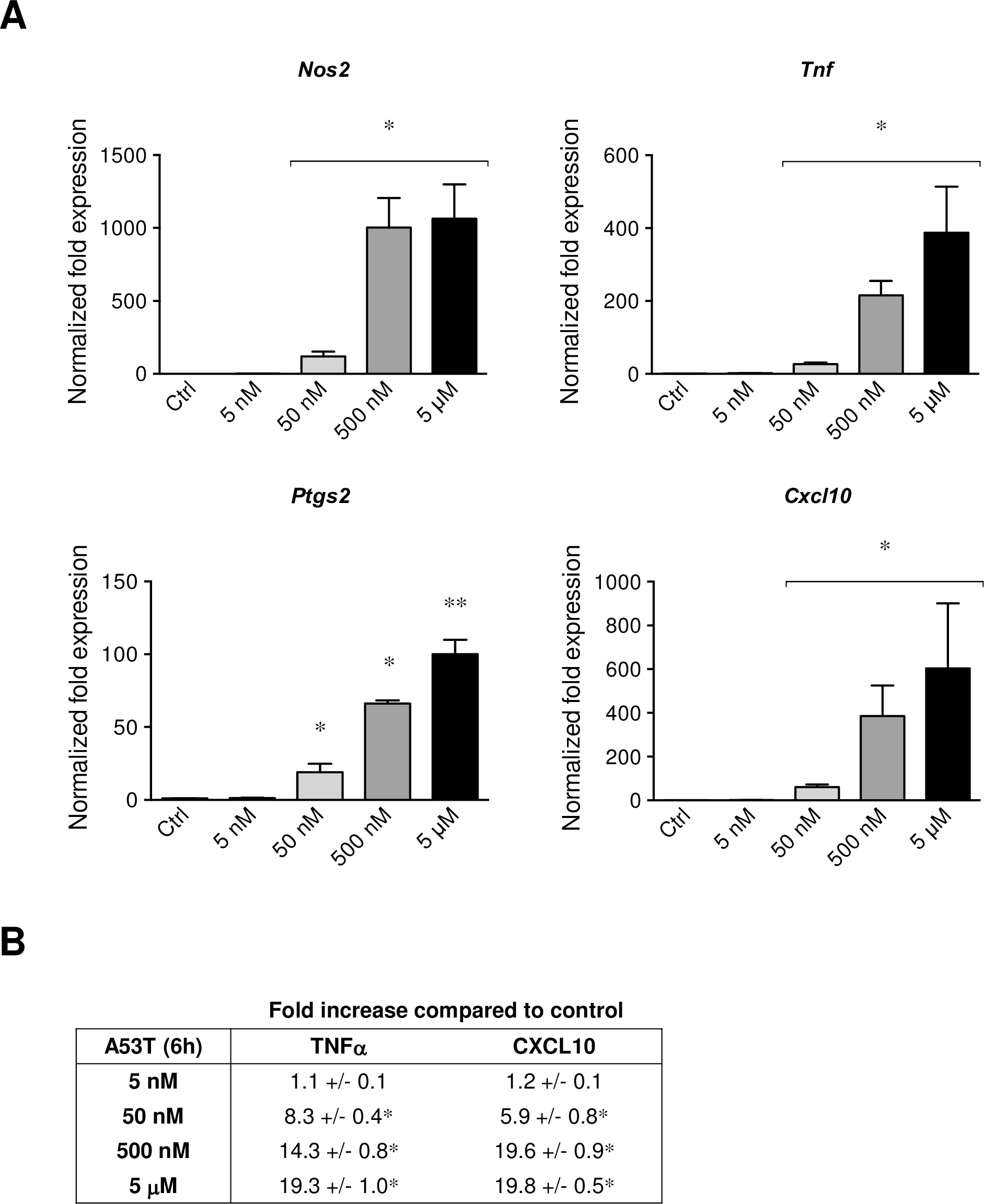

Supplement: S3 Fig — Following exposure of primary mouse microglial cells to different concentrations of A53T protein for 6 h, pro-inflammatory gene expressions (Nos2, Tnf, Ptgs2 and Cxcl10) were analyzed by real-time PCR (S3A Fig). Control expression levels were fixed at 1.0. All gene expressions were normalized to Rpl27 expression level. Results are given as mean ± SEM (n = 3 independent experiments). In these conditions, pro-inflammatory mediators released in the supernatant of microglial cultures were quantified by ELISA (S3B Fig). Results are expressed as fold increase compared to control and are given as mean ± SEM (n = 3 independent experiments). * p < 0.05, ** p < 0.01, significantly different from control condition. (TIF) [file pone.0162717.s003.tif]
